# Applying revised gap analysis model in measuring hotel service quality

**DOI:** 10.1186/s40064-016-2823-z

**Published:** 2016-07-28

**Authors:** Yu-Cheng Lee, Yu-Che Wang, Chih-Hung Chien, Chia-Huei Wu, Shu-Chiung Lu, Sang-Bing Tsai, Weiwei Dong

**Affiliations:** 1 Department of Technology Management, Chung-Hua University, Hsinchu, 300 Taiwan; 2 Department of Business Administration, Chung-Hua University, Hsinchu, 300 Taiwan; 3 PhD Program of Technology Management, Chung-Hua University, Hsinchu, 300 Taiwan; 4 Department of Business Administration, Lee-Ming Institute of Technology, Taipei City, 243 Taiwan; 5 Department of Tourism and Leisure Management, Yuanpei University of Medical Technology, Hsinchu, 300 Taiwan; 6 Zhongshan Institute, University of Electronic Science and Technology of China, Guangdong, 528402 China; 7 School of Economics & Management, Shanghai Maritime University, Shanghai, 201306 China; 8 Law School, Nankai University, Tianjin, 300071 China; 9 School of Business, Dalian University of Technology, Panjin, 124221 China; 10 School of Economics and Management, Shanghai Institute of Technology, Shanghai, 201418 China; 11 Department of Food and Beverage Management, Lee-Ming Institute of Technology, Taipei City, 243 Taiwan

**Keywords:** Service quality, HOLSERV, Gap analysis, Gap model, Business management

## Abstract

**Introduction:**

With the number of tourists coming to Taiwan growing by 10–20 % since 2010, the number has increased due to an increasing number of foreign tourists, particularly after deregulation allowed admitting tourist groups, followed later on by foreign individual tourists, from mainland China. The purpose of this study is to propose a revised gap model to evaluate and improve service quality in Taiwanese hotel industry. Thus, service quality could be clearly measured through gap analysis, which was more effective for offering direction in developing and improving service quality.

**Case description:**

The HOLSERV instrument was used to identify and analyze service gaps from the perceptions of internal and external customers. The sample for this study included three main categories of respondents: tourists, employees, and managers.

**Discussion and evaluation:**

The results show that five gaps influenced tourists’ evaluations of service quality. In particular, the study revealed that Gap 1 (management perceptions vs. customer expectations) and Gap 9 (service provider perceptions of management perceptions vs. service delivery) were more critical than the others in affecting perceived service quality, making service delivery the main area of improvement.

**Conclusion:**

This study contributes toward an evaluation of the service quality of the Taiwanese hotel industry from the perspectives of customers, service providers, and managers, which is considerably valuable for hotel managers. It was the aim of this study to explore all of these together in order to better understand the possible gaps in the hotel industry in Taiwan.

## Background

With the number of tourists coming to Taiwan growing by 10–20 % since 2010, the number has increased due to an increasing number of foreign tourists, particularly after deregulation allowed admitting tourist groups, followed later on by foreign individual tourists, from mainland China. Therefore, the tourism industry has become of greater economic importance, according to the Tourism Bureau statistics of Taiwan. The international tourist industry has experienced significant growth in recent years, and more and more hotels provide exquisite, high-quality and customized service that contributes to a hotel’s image and competitiveness in Taiwan (Chen 2013). Thence, the hotel sector within the tourism industry faces more intense global competition than other supply industries. Meanwhile, the rapidly growing number of visitors has increased the workload for hotel employees. Thus, they need to staff sufficient professional employees. However, the hotel industry may face the problem of finding qualified employees to provide services that could meet the standards of foreign tourists. Mei et al. (1999), Tsaur and Lin (2004) and Hooper et al. (2013) stated that one of the most influential factors on customers’ perceptions of service quality is the employees. Dedeoğlu and Demirer (2015) stress the factors contributing to hotel service quality are often the services related to employee behavior and tangibles. Tsang (2011) studied the Taiwanese hotel industry and found that success and failure in the service delivery of a hotel largely depends on the attitudes and behaviors of contact employees. Thus, determining how employees perceive the services they deliver becomes critical.

Service quality has been identified as crucial to the hotel industry and is measured to assist managers in making decisions, thus improving overall efficiency and profits. Service quality has gradually been recognized as a key factor in gaining competitive advantage and retaining customers (Callan and Kyndt 2001; Nasution 2016). Currently, Wu and Ko (2013) hotel organizations have difficulties in adequately assessing and improving their service performance from a customers’ perspective. They also fail to recognize which factors that customers consider important and when they should best evaluate their hotel experience. Moreover, while most of the studies on the hotel sector in the literature focus mainly on the evaluation of customers for service quality, other stakeholders’ (employees’ and managers’) perceptions have been ignored (Dedeoğlu and Demirer 2015).

Numerous empirical studies have shown that there were considerable differences in expectations of service quality between customers and management in the service industry (Tsang and Qu 2000; Kang and Bradley 2002; Lee et al. 2007; Chen and Chang 2005; Torres et al. 2013; Dedeoğlu and Demirer 2015). Tsang and Qu (2000) evaluated perceptions of service quality in China’s hotel industry, from the perceptions of both tourists and managers. Their results indicated that tourists’ perceptions of service quality were consistently lower than their expectations and managers overestimated the service delivery. Some studies have shown that front-line employees frequently serve on their way, so it is difficult for management to inspect their behavior (Bowen and Lawler 1992; Schneider and Bowen 1995; Yagil 2002). Dedeoğlu and Demirer (2015) addressed the nature and characteristics of differences in service quality perceptions among customers, managers and employees in the hotel industry. Moreover, Torres et al. (2013) emphasized that studies are required in the field that include the examination of various kinds of feedback (i.e. guests, experts, and operators). The different levels of value provide the need for tourism and hospitality operators to adopt a more comprehensive strategy to collect, analyze, and take appropriate actions. Little empirical research has existed on the evaluation of service quality from the perspective of managers, employees and customers in hotel industry in Taiwan. We believed that management should better understand the customers’ expectations that would influence design, development and delivery the service offering. Employees contact with customers should offer consistent quality of services that would attract and maintain customers directly. The evaluation of the service quality should not only base on customers and managers but also employees, consequently that it is able to assess customer needs and wants accurately. Hence, it is essential to understand the perceptions of customers in relation to the perceptions of managers and employees.

Although several researchers (e.g. Carman 1990; Teas 1993) have criticized Parasuraman and et al.’ (1985, 1988) gap analysis in measuring customer’s service quality perceptions and expectations, it is still the leading measure of service quality (Lam and Woo 1997). However, Gap 5 has functional relationships with Gaps 1–4 in the PZB model, these relationships are problematic due to the individual measurement of a gap cannot be determined by combining the gaps. Therefore, a number of researchers have revised the gap model to focus on Gap 5, Gap 1, and other additional gaps (Jannadi et al. 2000; Tsang and Qu 2000; Chen and Chang 2005; Kang and Bradley 2002; Dedeoğlu and Demirer 2015). Some researchers have confirmed that a revised gap model was relevant to the research scope and effectively evaluated service quality problems which could provide management with important insights. Particularly, Lee et al. (2007) revised the gap model by decomposing service activities and focused on Gap 5, Gap 1, and three identified additional gaps (Gap 8, Gap 9, and Gap 10). Through the revised gap model, Lee et al. (2007) stressed that service quality could be clearly measured through these gap scores, which were more effective for offering direction in developing and improving service quality. This study contributes toward an evaluation of the service quality of the Taiwanese hotel industry from the perspectives of customers, service providers, and managers, which is considerably valuable for hotel managers. Furthermore, the study of various sources of perspectives (i.e. tourists, managers, and employees) is often studied separately in the tourism literature. It was the aim of this study to explore all of these together in order to better understand the possible gaps in the hotel industry in Taiwan.

## Literature review

### Service quality literature

**Fig. 1:**
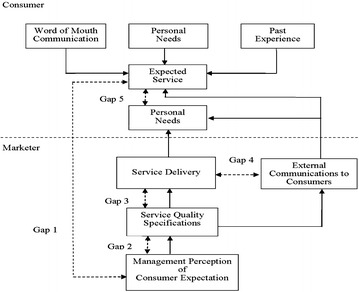
Service quality model

The SERVQUAL model is the most widely used instruments to measure the customer satisfaction in various industries and across different countries, developed by Parasuraman et al. 1985, then refined in 1988 and 1991. The model is based on the customer’s assessment of service quality, which is a comparison of the expected and the obtain value as well as a consideration of gaps in the process of service provision. The foundation of SERVQUAL instrument was the gap model. The model shown in Fig. 1 identifies five gaps. Gap 1 is the difference between customer expectation and management perceptions of customer expectation, Gap 2 is the difference between management perceptions of customer expectations and service quality specifications, Gap 3 is the difference between service quality specifications and the service actually delivered, Gap 4 is the difference between service delivery and external communication, and Gap 5 is the difference between customer expectation on the service and their perceptions of service performance.

Previous studies (Brown et al. 1993; Babakus and Boller 1992; Martin 2003; Han and Baek 2004; Gonzalez et al. 2008; Wei et al. 2011; Stefano et al. 2015) have applied SERVQUAL to measure Gap 5 and Gap 5 has functional relationships with Gaps 1–4 in the PZB model. However, these relationships are problematic because the individual measurement of a gap cannot be determined by combining the gaps.

### Application of the Gap model

Luo and Qu (2016) indicated quality of service is more difficult to define, measure, and manage than manufacturing products due to the unique characteristics of services.

Saleh and Ryan (1991) identified the existence of gaps between clients’ and management perceptions of attributes of the hotel, and between client expectation and perception of the services offered. Some researchers (Large and Konig 2009; Frederick and Mukesh 2001) designed INTSERVQUAL, an internal service quality measurement scale based on the “gap model” to successfully measure the difference between internal customers’ understanding and expectation from frontline service staff. Dedeoğlu and Demirer (2015) indicated while most of the studies on the hotel sector in the literature focus mainly on the evaluation of customers for service quality, other stakeholders’ (employees’ and managers’) perceptions have been ignored. It is argued that the existence of these gaps is a source of dissatisfaction with services provided (Saleh and Ryan 1991). Therefore, numerous of studies have been revised the gap model to focus on Gap 5, Gap 1, and other additional gaps (Jannadi et al. 2000; Tsang and Qu 2000; Chen and Chang 2005; Kang and Bradley 2002; Dedeoğlu and Demirer 2015). Jannadi et al. (2000) investigated four gaps of service quality in the Saudi Consolidated Electric Company in the Eastern Province and revealed that Gap 3 (service performance) was more critical than the others in affecting perceived service quality, making service delivery the main area of improvement. In addition, there was a revised gap model concentrated on Gap 5, Gap 1, and two additional identified gaps (Gap 6 and Gap 7) demonstrated by Tsang and Qu in 2000. Moreover, a conceptual “gaps model” of information technology (IT) service quality was developed by Kang and Bradly in 2002, which identified seven gaps between customers and IT service suppliers. Dedeoğlu and Demirer (2015) addressed the nature and characteristics of differences in service quality perceptions among customers, managers and employees. Torres et al. (2013) emphasized that studies are required in the field that include the examination of various kinds of feedback (i.e. guests, experts, and operators). The different levels of value provide the need for tourism and hospitality operators to adopt a more comprehensive strategy to collect, analyze, and take appropriate actions.

**Fig. 2:**
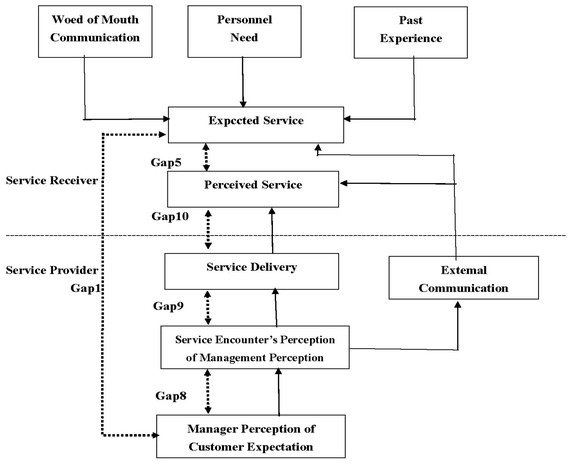
Revised conceptual model. Source: Adapted from Lee et al. (2007)

Some researchers have confirmed that a revised gap analysis was relevant to the research scope and effectively evaluated service quality problems which could provide management with important insights. Particularly, Lee et al. (2007) revised the conceptual model by decomposing service activities and focused on Gap 5, Gap 1, and three identified additional gaps (Gap 8, Gap 9, and Gap 10); Although Gap 5 has a functional relationship with Gaps 1–4 in the PZB model (Parasuraman et al. 1985), individual measurement of gap cannot be shown as the combination of gaps. Therefore, our studies applied the revised gap model designed by Lee et al. (2007). The structure is shown conveniently identified and service quality could be clearly measured through these gap scores in Fig. 2.

The definitions of Gap 5 and Gap 1 are the same as in the PZB model; Gap 5 is the difference between customer perceptions and expectations, and Gap 1 is the difference between management perceptions and customer expectations. Gap 8 is the difference between management perceptions of customer expectations and service encounter perceptions of management perceptions. Gap 9 is the difference between service encounter perceptions of management perceptions and service delivery. This gap represents the gap of service perceptions through the service delivery process. Gap 10 is the difference between service delivery and the perceived service. The functional relationship can be indicated as follows:

1 $${\text{Gap }}5 \, = {\text{ Gap }}1 \, + {\text{ Gap }}8 \, + {\text{ Gap }}9 \, + {\text{ Gap }}10$$

The revised conceptual model is another better way to measure gaps of service quality, because it provides a functional relationship that indicates the combination of gaps and the decomposition of service activity. Especially, it offers direction for developing and improving service quality as well (Lee et al. 2007).

### Service quality of an international hotel

Despite SERVQUAL’s wide use by academics and practicing managers in various industries, a number of studies have questioned the conceptual and operational base of the model (Babakus and Boller 1992; Carman 1990; Teas 1994; Saleh and Ryan 1991). Some researchers suggested that further customization of the scale for the hospitality industry was necessary (Saleh and Ryan 1991). Various measurement scales such as LODGSERV, HOLSERV, LODGQUAL and DINESERV have been developed for service quality evaluation purposes in the tourism industry. LODGSERV (Knutson et al. 1990) and HOLSERV (Mei et al. 1999) are used in the accommodation industry. LODGQUAL (Getty and Thompson 1994) is to assess service quality in the lodging industry while DINESERV (Stevens et al. 1995) is used in the restaurant services sector. Knutson et al. (1990) adapted SERVQUAL dimensions and developed an instrument called LODGSERV. Reliability is the most critical element in LODGSERV. By contrast, Kandampully and Suhartanto (2000) identified customer satisfaction with housekeeping as the only significant factor affecting customer loyalty. Mei et al. (1999) revised SERVQUAL (Parasuraman et al. 1991) to include three dimensions of service quality: employees, tangibles, and reliability. They found that the employee dimension was the best predictor of overall service quality. Dedeoğlu and Demirer (2015) stress the factors contributing to hotel service quality are often the services related to employee behavior and tangibles. In addition, HOLSERV scale more parsimonious and user-friendly than SERVQUAL (Wu and Ko 2013). Moreover, consideration of the type of hotel and the range of facilities available, the HOLSERV instrument is suitable for our study in the hospitality industry in Taiwan, to design service strategies that meet guest expectations.

## Methodology

### Questionnaire design and distribution

After a review of the literature, the HOLSERV instrument by Mei et al. (1999) was used to identify and analyze service gaps among the perceptions of tourists, employees and hotel managers. The gap in service quality was measured using the 27 items of the HOLSERV, with a 9-point scale ranging from 1 (completely unfulfilled) to 9 (much fulfillment). The sample comprised three main categories of respondents: tourists; employees, and managers. The first category of the questionnaire was designed to examine the tourists’ expectations and perceptions of service quality. The second category of the questionnaire was designed to evaluated managers’ perceptions of customer expectations. The third category of the questionnaire was designed to assess employee perceptions of manager perceptions and employee perceptions of perceived service. The target population of the tourist survey was all international tourists who visited hotels in Taipei, Taiwan were chosen for this study.

A total of 382 tourists were invited to complete the questionnaire, and 341 effective samples were obtained (usable response rate of 89.2 %). The gender breakdown of the respondents was 56.3 % male and 43.7 % female. Of the 341 respondents, 255 were tourists, 40 were managers, and 46 were employees.

### Demographic profile of the hotel tourists, employees, and managers

The questionnaire survey sites selected for this study were two international hotels in Taiwan. A convenience sampling method was applied. Ultimately, 300 tourists were invited to complete the questionnaire and 255 effective responses were obtained (for a usable response rate of 83.30 %). The sample of tourists contained more males (55.69 %) than females (44.31 %). More than half of the respondents had a university, college, or graduate education. Approximately 55 % of the respondents were professionals, executives, or sales people, and nearly 55 % earned an annual household income of US $32,000 or above. The majority of the respondents (60 %) were aged 21–40 years. Most of the respondents were from the cities of Taipei (55 %), Tainan (15 %), or Taichung (10 %), and the rest of the respondents (20 %) were from other countries.

The target population for the management survey was all supervisors and managers (ranging from the supervisor to the general manager level) who worked in two hotels located in the cities of Taipei and Taichung. The sample size was 40. The sample of managers contained more males (60 %) than females (40 %), and more than 80 % were aged 31–50 years. More than 60 % of the respondents had a university, college, or graduate education. The respondents ranged from supervisors to general managers, and 68 % were departmental managers or supervisors. More than a quarter of respondents worked in the housekeeping department, followed by the front desk (16 %), training (14 %), food and beverage (11 %) and other departments (30 %).

The target population for the employee surveys were from the housekeeping department, front desk, training, food and beverage, and other departments. The sample size was 46. The sample of employees contained more males (56.52 %) than females (43.48 %) and more than 70 % were aged 21–40 years. More than half of the respondents had a university, college, or graduate education.

## Results

### Gap 5

As noted in Table 1, the results of Gap 5 indicated that, all attributes were negative scores, and there was a significant difference between tourists’ actual perceptions and their expectations. And overall service quality provided below tourists’ expectation. The biggest gaps were on attributes, 6 “Gives prompt service (−1.048)”, 4 “Provides services at the time it promises to do so” (−0.932), 1 “Promises to provide a service and does so” (−0.915), 2 “Shows dependability in handling service problems” (−0.915), and 3 “Performs the service right the first time” (−0.881). Those attributes were the most serious deficiencies which would need pay close attention by managers and make improvement effectively. The overall Gap 5 score was −0.662 which would showed that the overall service quality provided by the hotel in Taiwan would not meet tourists’ expectation.

**Table 1:** Revised gap scores and functional relationships (Gap 5 = Gap 1 + Gap 8 + Gap 9 + Gap 10)

Question	Gap 5 TP-TE	Gap 1 MPTE-TE	Gap 8 EPMR-MPTE	Gap 9 EPD-EPMR	Gap 10 TP-EPD
1. Promises to provide a service and does so	−0.915	−1.036	0.767	−0.696	0.050
2. Shows dependability in handling service problems	−0.915	−0.668	0.383	−0.630	0.000
3. Performs the service right the first time	−0.881	−0.891	0.460	−0.500	0.050
4. Provides services at the time it promises to do so	−0.932	−0.377	−0.077	−0.478	0.000
5. Tells guests exactly when the services will be performed	−0.770	−0.555	0.107	−0.522	0.200
6. Gives prompt service	−1.048	−0.750	0.174	−0.522	0.050
7. Always willing to help	−0.811	−0.514	0.125	−0.522	0.100
8. Never too busy to respond to guests’ requests	−0.611	−0.877	0.466	−0.500	0.300
9. Instils confidence in guests	−0.610	−0.318	0.036	−0.478	0.150
10. Guests feel safe in the delivery of services	−0.698	−0.500	0.174	−0.522	0.150
11. Guests feel safe and secure in their stay	−0.648	−0.323	−0.053	−0.522	0.250
12. Polite and courteous employees	−0.626	−0.509	0.170	−0.587	0.300
13. Have the knowledge to answer questions	−0.741	−0.559	0.018	−0.500	0.300
14. Have the skill to perform the service	−0.515	−0.736	0.528	−0.457	0.150
15. Gives individual attention	−0.520	−0.650	0.239	−0.609	0.500
16. Deals with guests in a caring fashion	−0.660	−0.500	0.283	−0.543	0.100
17. Has guests’ best interests at heart	−0.448	−0.268	0.144	−0.674	0.350
18. Understands guests’ specific needs	−0.783	−0.591	0.243	−0.435	0.000
19. Equipment, fixtures and fittings are modern looking	−0.451	−0.814	0.559	−0.496	0.300
20. Facilities are visually appealing	−0.241	−0.427	0.445	−0.609	0.350
21. Neat and professional employees	−0.239	−0.518	0.644	−0.565	0.200
22. Materials are visually appealing	−0.842	−0.873	0.403	−0.522	0.150
23. Fixture and fittings are comfortable	−0.563	−0.514	0.494	−0.543	0.000
24. Equipment and facilities are easy to use	−0.547	−0.468	0.362	−0.391	−0.050
25. Equipment and facilities are generally clean	−0.558	−0.518	0.427	−0.717	0.250
26. Variety of food and beverages meet guests’ needs	−0.628	-0.464	0.516	−0.630	−0.050
27. Services are operated at a convenient time	−0.694	−0.518	0.383	−0.609	0.050
Average	−0.662	−0.583	0.312	−0.547	0.156

*TE* tourist expectation, *TP* tourist perception, *MPTE* managers’ perception for tourist s’ expectation, *EPMR* employees’ perception for managers’ requirement, *EPD* employees’ perception for delivery

### Gap 1

As shown in Table 1, a comparison of managers’ perception for tourists’ expectation and the tourists’ themselves expectation. The result indicted that, the overall Gap 1 score was −0.583, which would indicated that managers do not have a good understanding of tourist expectation. This finding contrasts with previous studies (Nel and Pitt 1993; Tsang and Qu 2000) but consistent with past research (Choy et al. 1986; Wei et al. 1989).

All 27 attributes were negative and very big. The range from attribute 17 “Has guests’ best interests at heart” (−0.268) to attribute 1 “Promises to provide a service and does so” (−1.036) was big variation. The biggest gaps were on attributes, 1 “Promises to provide a service and does so” (−1.036), 3 “Performs the service right the first time” (−0.891), 8 “Never too busy to respond to guests’ requests” (−0.877) and 22 “Materials are visually appealing” (−0.873). Therefore, from the results of negative Gap 1 score and big difference, it can be concluded that Gap 1 tend to a major problems related to Gap 5 of service quality in the hotel case.

### Gap 8

As noted in Table 1, for the most part, employees’ perception for delivery are more than managers’ perception for tourists’ expectation except attribute 4 “Provides services at the time it promises to do so” (−0.077) and attribute 11 “Guests feel safe and secure in their stay” (−0.053). In addition, the overall Gap 8 score was +0.312, which would indicted that the managers tend to have good communication with employees for understanding tourists’ expectation. Hence, Gap 8 is probably not to be a major problem of service quality in the hotel case.

### Gap 9

As shown in Table 1, the mean score gaps along each of 27 attributes was calculated for employees’ perception of manager’s requirement and service delivery by themselves. The result of the overall Gap 9 score for this study was −0.547 and all difference of attributes were negative and very big. The range from attribute 24 (−0.391) to attribute 25 (−0.717), was quite big variation. The biggest gaps were on attributes, 25 “Equipment and facilities are generally clean” (−0.717), 1 “Promises to provide a service and does so” (−0.696), 17 “Has guests’ best interests at heart” (−0.674), 2 “Shows dependability in handling service problems” (−0.630) and 26 “Variety of food and beverages meet guests’ needs (−0.630)”. Accordingly, from the results of negative Gap 9 score and big difference, it also can be concluded that Gap 9 seems one of major problems related to Gap 5 of service quality in the hotel case.

### Gap 10

The results of Table 1 show that, for most part, employees believed that their perception for delivery are more than tourists themselves perception except attributes 24 “Equipment and facilities are easy to use” (−0.050) and attribute 26 “Variety of food and beverages meet guests’ needs” (−0.050). In addition, the overall gap 10 score was 0.156, which would indicated that the employees tend to have a good understanding of customer expectations. Therefore, from the results of positive gap 10 score, consequently, Gap 10 did not seem to a major problem of service quality in the hotel case as well.

## Discussion

This study provided a new measurable instrument and expressed the evaluation results of the service quality gap between expectations and perceptions for tourists, managers, and employees in the hotel industry. Thus, this study identified the gaps (Gap 5, Gap 1, Gap 8, Gap 9, and Gap 10) that could appear from inconsistency in the expectations and perceptions of service quality among tourists, management, and employees and demonstrated how the gaps could be reduced.

An analysis of Gap 5 illustrated how the gaps between customers’ perceptions of service quality and their expectations could be reduced. The Gap 5 analysis indicated that tourists’ perceptions were consistently lower than their expectations. The overall Gap 5 score was −0.662, which showed that the overall service quality provided by the hotel industry in Taiwan was below tourists’ expectations. According to our Gap 5 analysis, the biggest gaps were associated with “gives prompt service,” “provides services at the time it promises to do so,” and “promises to provide a service and does so.” This indicates a problem of reliability and responsiveness in service quality. The negative Gap 5 scores clearly showed that managers in the Taiwanese hotel industry must still improve and enhance its service quality. The Gap 5 analysis was essential because it offered a measurable and useful tool for the management to identify the service problems in the hotel industry in Taiwan. In additional, managers should consistently implement such analysis so that they can further understand the tourists’ evaluation process and their consumer experiences and hence meet their expectations more consistently. However, to reduce Gap 5, managers should also concern themselves with the other four gaps (Gap 1, Gap 8, Gap 9, and Gap 10) that contribute to Gap 5. Therefore, this revised gap model offers a method for managers to identify the causes of Gap 5 that can be clearly measured through the gap scores of the hotel industry in Taiwan. According to our results, the functional relationship can be expressed as follows:

2 $${\text{Gap }}5( - 0.662) = {\text{ Gap }}1( - 0.583) + {\text{ Gap }}8( + 0.312) + {\text{ Gap }}9( - 0.547) + {\text{ Gap }}10( + 0.156)$$

Examining Gap 1 was a necessary step that contributed toward the understanding of whether managers accurately perceive tourists’ service quality expectations from the Taiwanese hotel industry. The result showed that the overall Gap 1 score was −0.583, indicating that managers do not fully understand customer expectations. Accordingly, given the negative Gap 1 score results and the large difference in expectations and perceptions, we conclude that Gap 1 is one of the major problems of service quality and that it contributes to Gap 5. Moreover, when managers’ perception of tourists’ expectations is close to the tourists’ expectations (Gap 1), the difference in customers’ perceptions of service quality and their expectations can be narrowed (Gap 5) as well. Our findings are consistent with those of past studies (Coyle and Dale 1993; Zeithaml et al. 1990; Tsang and Qu 2000) that have argued that managers traditionally have the least contact with customers and are thus unable to understand customer wants accurately. Thus, they might initiate a chain of bad decisions, leading to poor perceived service quality. In improving the service quality (i.e., narrowing Gap 5), managers should re-examine the service delivery process that meets tourists’ requirements and wants in the Taiwanese hotel industry. The hotel management should attempt to address marketing research orientation, upward communication, and the quality of management. To gain first-hand knowledge of tourists’ expectations and perceptions, senior management should consistently contact tourists and inquire about the actual service delivery. Thus, managers can more accurately fulfill tourists’ expectations and provide the desired level of service performance.

Assessing Gap 8 was a critical task that contributed toward knowing whether employees accurately perceive tourists’ service quality expectations from the Taiwanese hotel industry. The overall Gap 8 score was 0.312, indicating that the managers did a good job at training or communicating with employees to understand customers’ expectations. Hence, Gap 8 was not a primary contributor to Gap 5.

Evaluating Gap 9 was crucial to identifying whether employees followed managers’ requirements and were able to perform services at the desired level in the Taiwanese hotel industry. The Gap 9 score in this study was −0.547, indicating a difference between service performance standards and the actual service delivered. Gap 9 often occurred because of some limits, such as poor service attitudes, poorly qualified employees, insufficient service capacity, and inadequate internal communication systems. Because of these constraints, employees could not offer services at the level required by the management. The existence of Gap 9 was related to Gap 1. Moreover, if managers do not fully understand tourists’ expectations, employees cannot deliver service adequately. Therefore, to reduce the gap between employees’ perceptions of managers’ requirements and service delivery, managers in the hotel industry in Taiwan should apply internal investigation systems to evaluate whether their employees can meet the stipulated service standards.

Measuring Gap 10 contributed toward assessing whether employees overestimate whether their service delivery meets tourists’ expectations. The overall score in Gap 10 for this study was 0.156, which indicated that employees tended to have a reasonably good understanding of customer expectations. Because of the positive score results, Gap 10 was deemed not to be a primary contributor to Gap 5.

## Conclusion

This research makes the following three contributions. First, this research develops an evaluation of the service quality of the Taiwanese hotel industry from the perspectives of customers, service providers, and managers, which is considerably valuable for hotel managers. Second, this study explores all of perspectives (i.e. tourists, managers, and employees) together in order to better understand the possible gaps in the hotel industry in Taiwan. This revised gap analysis model can be the reference for related research. Third, the study revealed that Gap 1 (management perceptions vs. customer expectations) and Gap 9 (service provider perceptions of management perceptions vs. service delivery) were more critical than the others in affecting perceived service quality, making service delivery the main area of improvement.

The results of this study also provide a number of managerial contributions. Managers should understand the reason why these differences occur, suggesting that management in the hotel industry spend more time interacting with tourists and conduct internal investigations to assess if their employees are able to meet the service standards, so that the employees willingly provide a good quality of service that benefits hotel operations. Moreover, with the revised gap analysis, managers can effectively prioritize a task to effectively compensate for shortfalls in the provided service. Obviously, managers should eliminate the existence of Gap 1; subsequently, Gap 9 could be reduced. Likewise, if managers do not have a clear perception of customer expectations, employees cannot deliver adequate services. To improve service quality efficiently, managers must be able to identify the priorities of improvements of service attributes especially with limited resources. Finally, the revised gap analysis enable managers to understand the specific attributes that significantly affect service quality and thus enables them to recognize the explicit quality attributes that must be improved and would enhance customer satisfaction within the hotel industry. Overall, this study not only proposes reliable and effective methods but also obviously recognizes which factors that customers consider important as related to management when they should best evaluate their hotel experience. Applying reliable and effective methods for service quality improvement, will lead to a higher level of customer satisfaction and profitability of a firm. In summary, Travel and Tourism is an important economic activity in most countries around the world which not only affect the economic growth but also to increase employment opportunities.

## Limitations and suggestions for future research

There are some limitations in this study that must be recognized. First, the international tourist hotels surveyed in this study were small- and medium-sized hotels operating in Taipei. Because Taipei is more modern and well developed than are some other Taiwanese cities, these results might not represent the quality of hotel services across Taiwan. Second, the sample size was quite small (N = 255), employees (N = 46), and managers (N = 40). Future research should collect a larger number of samples and include a more diverse range of tourists, employees, and hotel managers. Third, this study conducted preliminary research into hotel services. Thus, the findings cannot be generalized to other service sectors. Future studies should collect data from different industries, such as banks, airlines, insurance providers, and call centers, to extend the scope of our findings. Fourth, this research was only limited to three-star hotels in Taiwan. Future studies should attempt to examine service quality across different hotel ratings or countries. This may provide an opportunity to compare the quality of service based on different hotel ratings (e.g., four or five-star hotels) or countries. Likely, Luo and Qu (2016) indicated that Westerners were more satisfied with service quality than do Chinese guests. In addition, this research could be applied to different categories, for example three-star, aparthotels, motels, inns and boutique. Finally, the findings of this study were based on a survey. Hence, future studies should apply a qualitative design to obtain an in depth understanding of the perceptions of customers in relation to those of managers and employees.
